# Early introduction is not enough: CSACI statement on the importance of ongoing regular ingestion as a means of food allergy prevention

**DOI:** 10.1186/s13223-023-00814-2

**Published:** 2023-07-18

**Authors:** Elissa M. Abrams, Moshe Ben-Shoshan, Jennifer L. P. Protudjer, Elana Lavine, Edmond S. Chan

**Affiliations:** 1 Department of Pediatrics, Section of Allergy and Clinical Immunology, University of Manitoba, FE125-685 William Avenue, Winnipeg, MB R3E 0Z2 Canada; 2 Department of Pediatrics, Division of Allergy and Immunology, University of British Columbia, 4480 Oak Street, Vancouver, BC V6H 3V4 Canada; 3 Division of Allergy Immunology and Dermatology, Department of Pediatrics, Montreal Children’s Hospital, McGill University, Montreal, Canada; 4 Department of Pediatrics and Child Health, Max Rady College of Medicine, Rady Faculty of Health Sciences, University of Manitoba, Winnipeg, MB Canada; 5 Children’s Hospital Research Institute of Manitoba, Winnipeg, MB Canada; 6 Department of Food and Human Nutritional Sciences, Faculty of Agricultural and Food Sciences, University of Manitoba, Winnipeg, MB Canada; 7 George and Fay Yee Centre for Healthcare Innovation, Winnipeg, MB Canada; 8 Institute of Environmental Medicine, Karolinska Institutet, Stockholm, Sweden; 9 Department of Pediatrics, University of Toronto, Toronto, Canada; 10 Department of Pediatrics, Queen’s University, Humber River Hospital, Toronto, ON Canada; 11 Division of Allergy, Department of Pediatrics, University of British Columbia, BC Children’s Hospital, Vancouver, BC Canada

Both randomized controlled and observational studies demonstrate a reduction in the risk of food allergy with early introduction of common allergens, in particular peanut and egg [1–4]. While these studies vary in design, population, dose and allergen used, there is a strong and consistent trend that early introduction has a role in the prevention of food allergy. As a result, guidelines clearly and consistently recommend early allergen ingestion (commonly operationalised as between ages 4–6 months), often in particular for higher risk populations, as a means of food allergy prevention [3, 5]. The current Canadian Society of Allergy and Clinical Immunology/Canadian Pediatric Society position statement recommends introduction of common allergens in high risk infants (with eczema or an immediate family history) at around 6, but not before 4 months of age when they are developmentally ready.In lower risk infants, introduction should be at around 6 months of age [5].

However, despite changes in guidance, recent epidemiologic evidence suggests that early introduction alone is not sufficient to reduce peanut allergy prevalence. In Australia, where uptake of peanut introduction in the first year of life increased more than threefold with adoption of early introduction guidelines, Soriano et al. reported observational data that peanut allergy prevalence had not significantly changed between 2007 and 2018 (3.1% to 2.6%, respectively; difference − 0.5% [95%CI  − 1.4%to 0.4%]; p = 0.26). This suggests that additional factors continue to play a role in the development of peanut and other food allergy (although with the guidelines only changing in 2016 studies may need longer to completely address this) [6].

While most Australian families were introducing peanut in infancy, Soriano reported that only ~ 30% of infants were eating peanut 2 or more times a week. A substantial proportion were eating peanut less than once a week and some had even eaten peanut only once (a bite or taste). This suggests that a lack of regular ingestion may be a key reason for the lack of change in prevalence despite early introduction [7]. Although most allergy prevention guidelines, including the current CSACI/CPS guideline, recommend ongoing regular ingestion as a means of food allergy prevention [5, 8], this guidance is often not translated into practice, or individual recommendations to parents. Therefore, the goal of this statement is to emphasize that current best evidence supports the importance of regularity of infant allergen ingestion, operationalised as at least once weekly, as a means of food allergy prevention.

Immunologically, the premise of the “dual-allergen exposure hypothesis” is based on the need for oral tolerance to supersede cutaneous (or possibly respiratory) sensitization, and mechanistically this implies that consistent, regular oral ingestion is necessary to induce and maintain tolerance [9, 10]. Recent animal data suggests that regular peanut ingestion induces a regulatory T-cell population that express high levels of CTLA-4, which in turn suppresses T follicular helper cells and germinal center B-cells induced by environmental peanut exposure [11, 12]. All clinical studies on food allergy prevention have suggested both early introduction and ongoing regular ingestion to achieve optimal food allergy prevention. For example, in the Learning Early About Peanut (LEAP) study, infants in the early introduction group were introduced to peanut at age 4–11 months but also ate peanut at least 3 times a week (total of 6 g, or the equivalent of about 24 peanuts, per week) until age 5 years compared to strict avoidance [1]. Similarly, in the PETIT randomized controlled trial that compared early (age 6 months) vs delayed (age 12 months) egg introduction in infants with atopic dermatitis, the early introduction arm included ongoing feeding of egg at least daily [4]. Observational studies on early food introduction also support ongoing regular ingestion. For example, Katz et al. reported a large observational study in which delayed (after 14 days) and/or irregular (< 1/day) cow’s milk ingestion significantly increased the risk of cow’s milk allergy (OR 19.3) compared to introduction in first 14 days of life [13]. In another case–control study of children with confirmed cow’s milk allergy (compared to nonatopic controls and children with egg allergy), irregularity of ingestion (< 1/day) increased the risk of cow’s milk allergy [14]. A recent large prospective interventional study in which 1992 newborns were recruited shortly before birth to either exclusive breastfeeding or at least one meal of cow’s milk formula (with or without breastfeeding) daily for the first 2 months of life found a significant reduction in cow’s milk allergy at 12 months (1.58% in the breastfed group compared to 0% in the other groups; relative risk 29.98, p < 0.001) with regular cow’s milk formula ingestion. The vast majority of those who developed cow’s milk allergy while being breastfed were exposed to small amounts of cow’s milk formula during the first 2 months of life (prevalence of 0.7% in the per-protocol exclusively breastfed group versus 3.27% among infants exposed to small amounts of cow’s milk formula), resulting in the authors’ conclusion that early continuous exposure should be encouraged, while occasional exposure increases risk of IgE-mediated cow’s milk allergy and should be avoided [15]. Based on the cow’s milk literature, the CSACI/CPS position statement currently recommends that intermittent supplementation with intact cow's milk formula should be avoided due to increased risk of cow's milk allergy. While there is less evidence for allergens other than cow’s milk, egg and peanut, the mechanism of sensitization is felt to be the same for all common allergens and hence regularity of ingestion is likely to be beneficial for other common allergens including tree nuts, sesame, grains and seafood.

The randomized controlled trials and observational studies regarding early ingestion of allergens have focused on the infant population, but there are now emerging data in older children (in particular those at risk of allergy such as siblings of peanut allergic children) that irregularity of ingestion may also increase the risk of food allergy [16]. A dose-dependent relationship between frequency of peanut ingestion and reduced risk of peanut allergy was reported among siblings of peanut allergic children. These siblings had tolerated oral peanut challenges at baseline, and during a median 2.9 years of follow up, none (0%) of the siblings who ate peanut at least once a month had peanut allergy vs 3% who ate it less than once a month and 18% who completely avoided peanut [16].

Once an allergen is ingested, the exact frequency of allergen ingestion required to maintain tolerance is not yet clearly established. For example, in a recently published cluster-randomized trial, general population infants introduced to peanut, cow’s milk, wheat and egg from 3 months of age (but only eaten once a week; and less so after 6 months of age) had a significantly lower risk of food allergy than those introduced at standard age (no intervention) [17]. However, given the above updated evidence, a pragmatic recommendation is for the relevant allergen/s to be consumed multiple times per month (with a goal of at least once each week), integrated into a family’s diet. The amount of allergen required for ingestion also remains undetermined, although a secondary analysis of the Enquiring About Tolerance (EAT) randomized controlled trial suggested a dose of about 2 g each of egg white and peanut protein per week (the equivalent of approximately one small boiled egg and 1.5 tsp peanut butter, respectively) is likely an amount that would be tolerated in infancy although further research is required and it is possible that smaller amounts may be effective as well [4, 18]. The long term duration of ingestion is unclear, but the LEAP-On study showed that when peanut was introduced in the 1 year of life and continued until 5 years of age, a subsequent 12 months of avoidance did not increase the prevalence of peanut allergy [19]. Based on this study, roughly 5 years of regular ingestion starting in infancy is likely a sufficient duration to induce long term tolerance.

In conclusion, our recommendations and considerations on frequency of ingestion are as follows (Fig. 1):

**Fig. 1:**
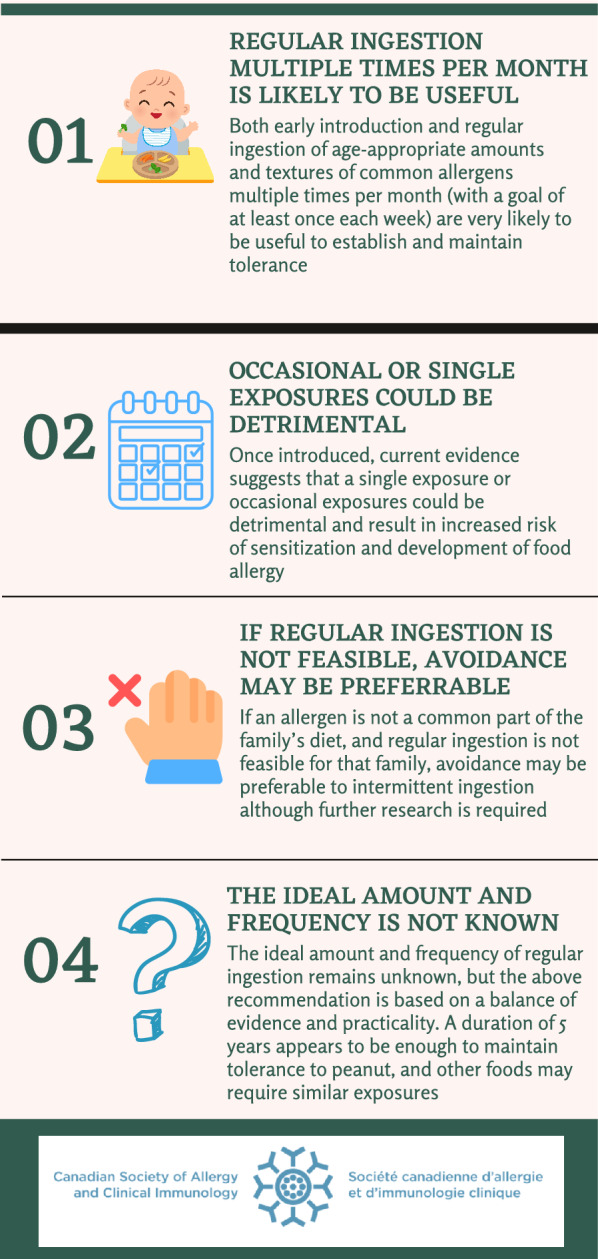
The importance of ongoing regular ingestion as a means of food allergy prevention

Recommendation:

1. Both early introduction and, once introduced, regular ingestion of age-appropriate amounts and textures of all common allergens multiple times per month (with a goal of at least once each week based on expert opinion) are very likely to be useful to establish and maintain tolerance.

Considerations:

- Once introduced, current evidence suggests that a single exposure or occasional exposures could be detrimental and result in increased risk of sensitization and development of food allergy.
- If an allergen is not a common component of the family’s diet, and regular ingestion is not feasible for that family, avoidance may be preferable to intermittent ingestion although further research is required.
- For newborn infants, exclusive breastfeeding is best, but if cow’s milk formula is introduced it should be given regularly (e.g. one feeding per day to supplement breastfeeding) from that point forward rather than intermittently to prevent cow’s milk allergy.
- The ideal amount and frequency of regular ingestion remains unknown, but the above recommendation is based on a balance of evidence and practicality. In general an age-appropriate serving could be aimed for. A duration of 5 years appears to be enough to maintain tolerance to peanut, and other foods may require similar exposures.

## Data Availability

Not applicable.
